# Enhancing *Vicia faba*^’^s immunity against *Rhizoctonia solani* root rot diseases by arbuscular mycorrhizal fungi and nano chitosan

**DOI:** 10.1186/s12870-023-04407-4

**Published:** 2023-08-24

**Authors:** Nashwa El-Gazzar, Kamar. M. Abd El-Hai, Safaa. A. M. Teama, Gamal. H. Rabie

**Affiliations:** 1 Department of Botany and Microbiology, Faculty of Science, Zagazig University, Zagazig, 44519 Sharkia Egypt; 2 Plant Pathology Research Institute, Agric., Res., Cent, Giza, Egypt

**Keywords:** *Rhizoctonia solani*, Mycorrhiza, Chitosan nanoparticles, Faba bean, Peroxidase, Poly phenol oxidase, Biological control

## Abstract

**Background:**

The spreading of root rot disease of faba bean plant (*Vichia faba* L, VF) in Egypt is still of great challenge faced researchers since VF is an important legume in Egypt, because their seeds are used for human feeding. Fungicides are used for treatment of either seeds or soil; unfortunately they cause environmental pollution. Therefore, there is a need to continue research to find out safe natural solutions. In this regard, Arbuscular mycorrhizal fungi (AMF) and chitosan (micro or nanoform) were used as an inhibitory product against *Rhizoctonia solani* OM918223 (*R.solani*) either singly or in combinations.

**Results:**

The results employed herein have exhibited that *R.solani* caused root rot disease of VF plants in more than 80% of the plants under investigation. Chitosan nanoparticles (Chitosan NPs) were prepared by ionic gelatin method and characterized by using dynamic light scattering (DLS), transmission electron microscopy (TEM) imaging and Fourier transform infra-red (FTIR). Chitosan NPs are spherical with a diameter of 78.5 nm and exhibited the presence of different functional groups. The inhibitory natural products against *R.solani* were arranged according to their ability to inhibit the pathogen used in the following descending manner; combination of AMF with Chitosan NPs, AMF with micro chitosan and single AMF, respectively. Where, Chitosan NPs showed a potent influence on *R.solani* pathogen and reduced the pre-and post-emergence of *R. solani*. In addition, Chitosan NPs reduced Disease Incidence (DI %) and Disease Severity (DS %) of root rot disease and are widely functional through mixing with AMF by about 88% and 89%. Further, Chitosan NPs and micro chitosan were proved to increase the growth parameters of VF plants such as nutritional status (mineral, soluble sugar, and pigment content), and defense mechanisms including total phenol, peroxidase, and polyphenol oxidase in mycorrhizal plants more than non-mycorrhizal one either in infected or healthy plants. Moreover, activity of AMF as an inhibitory against *R.solani* and improvement natural agent for VF growth parameters was enhanced through its fusing with Chitosan NPs.

**Conclusions:**

The use of AMF and Chitosan NPs increased faba bean plant resistance against the infection of root rot *R. solani*, with both prevention and cure together. Therefore, this research opens the door to choose natural and environmental friendly treatments with different mechanisms of plant resistance to disease.

## Highlights

Maintaining the good production and protection of VF plant against root rot disease was by either single AMF or in combination together with Chitosan NPs in order to limit losses.

The colonization of AMF over with Chitosan NPs exhibited more efficiency in inducing the VF resistance against *R.solani* than individual colonization of AMF alone.

The uses of AMF and chitosan derivatives either in normal or in nano form induce the VF plant to resist root rot disease.

It is an important to recall the positive side of Chitosan NPs was also restraint on AMF not on VF itself.

A perfect realization of the reaction among Chitosan NPs and AMF efficiency with the response of VF plant would open the door to select natural and eco-friendly treatments with different mechanisms of plant resistance to disease.

## Background

*Vicia Faba*.L (VF) is a major serious food and feed legume worldwide, which belongs to Fabaceae Family [1]. Root rot disease is one of the major limiting biological stress; that influence the improvement and productivity of VF. At the physiological scale, root rot disease commonly caused by diverse soil-borne fungi as *Rhizoctonia solani (R.solani), Fusarium solani, F. oxysporum, Sclerotinia spp.* and *Pythium sp* [1, 2]. In Saskatchewan, root rot disease affected the VF and decreased crop productivity by 60% [3]. In addition, according to Lamari and Bernier [3], VF root rot in Manitoba and Alberta was linked to *Rhizoctonia solani*. According to studies conducted from 2004 to 2006 [4], root rot was the most significant disease affecting VF production in central Alberta. Root rot infections damaged 40% of VF fields in 1978 and 70% of fields in 1979 [5]. According to Merga et al. [6], VF plantings covered 5–6 million hectares worldwide in 1961–1962 and produced 5 million tons of seeds. Following that, VF production fell, reaching two million acres and three million tons in 1990. Then, from 1991 to 2017, the area under cultivation for VF stayed the same, at around two million ha, while the yield decreased by 3–5 million tons. In Egypt, according to FAO [7], VF production has declined from 246,801 tons in 2008 to 119,104 tons in 2016 and the planting area decreased from 71.445 hectare in 2008 to 34.314 hectare in 2016 due to root rot and other fungal diseases. This showed that there is a need to continue research to find out effective products to control root rot disease.

Fungicides are utilized as control agents against fungal diseases as rizolex T.50wp, cyproconazole, hexaconazole, prodione, triazoles, phenylpyrroles, phenylamides, benzimidazoles, and strobilurines [8]. Occasionally, they give good results but they raise diverse issues as carcinogenicity, phytotoxicity and inhibitory effect on root nodule bacteria [9, 10]. According to Wyszkowska and Kucharski [11], frequent applying of fungicides has a threat to the environment because it increases the buildup of toxic substances in ecosystems and has a detrimental effect on soil microorganisms. For instance, copper fungicide inhibits photosynthesis, which has a negative impact on photosystem activity, the environmental balance and public health [12–14].This is another challenge to use hazardous and safe natural products to control root rot disease using mycorrhiza and nanoparticles. In this regard, Arbuscular mycorrhizal fungi (AMF) and chitosan nanoparticles were used as a trial to allow protection of VF against Rhizocotinia root rot disease.

Arbuscular mycorrhizal fungi (AMF) act as bio-control agent by indirect effect or direct on pathogen through host nutritional effects or enhancement plant defense resistance by accelerating nutrient absorption and activating antioxidant enzyme efficacy [15–17]. During root colonization by AMF, the host plant undergoes significant physiological changes that have an impact on interactions with a variety of below- and above-ground species. Several plant species, including significant crop kinds from the perspective of agriculture, have been shown to benefit from the symbiosis’ defense mechanisms against infections, pests, and parasitic plants. In addition, colonization by AMF induces a significant increase of the plant immune responses. This activation puts the plant in a primed condition that makes it possible for defense mechanisms to be activated more quickly in response to an attack from prospective foes [16]. The symbiosis of both VF plant with AMF can induce the plant tolerance to different environmental stress through the hyphae of AMF that accelerated the absorption area of the plant root and hence increasing nutrient and water absorption causing improvement in plant growth [15]. AMF releases enzymes, which allow them to digest and penetrate substrates, as well as some AMF produce phytohormone as indole-acetic acid, gibberellin, zeatin and abscisic acid that have direct inductions on plant propagation and physiological efficacy which improve the soil fertility and in turn plant growth [18, 19]. Additionally, by competing for nutrients and space, AMF might directly affect the pathogens. AMF has primary access to carbon through its host’s photosynthetic carbon as well as carbon compounds taken up by roots, where they compete for it. The physiological requirements are the same when microbes compete with one another for resources or space. Competition between soil-borne fungal diseases and AMF for root tissues is a possibility. A mature AMF colonization that is defined by the presence of arbuscules appears to be a requirement for biocontrol [20, 21].

Nanomaterials are eco-friendly substitutes which inhibit plant pathogens. Nanoparticles were showed to inhibit fungal pathogens than metal in micro-sized form. Antifungal activities of nanoparticles will be useful in soil as their developing management strategies to control pathogenic fungal disease and could be applicable in the field [22.23]. In the previous studies, the assimilation of some plants to chitosan nanoparticles increased their resistance to plant pathogenic fungi as Dematophora necatrix, Colletotrichum gloeosporioides, and Fusarium oxysporium compared to the control plants [1, 22]. Chitosan NPs refers to chitosan that has been processed into nanoparticles, which increases its potency and potential uses [23]. Chitosan and Chitosan NPs can transport biological controls like helpful bacteria or plant extracts with antimicrobial characteristics Chitosan and Chitosan NPs are useful instruments in plant protection and agriculture because to their established preventative and stimulating activities against plant diseases. They have the potential to be environmentally benign, sustainable solutions for managing plant diseases. Additionally, both dicotyledons and monocotyledons may effectively defend themselves against diseases using chitin and its derivative chitosan [23, 24]. Chitosan has antibacterial characteristics that are effective against a variety of bacterial, fungal, and viral plant diseases [25]. Chitosan NPs can have even more potent antimicrobial effects due to their increased surface area [23]. Hassan and Chang [26] explained the role of chitosan and Chitosan NPs in increasing plant defense and tolerance response due to the electrostatic action among amino acid sets in chitosan with microbial cell membranes, which leads to a change in the intracellular structure and paralysis in the microbe cell membranes that lead to death of plant pathogens. In addition, Chitosan NPs induced plant resistance against pathogenic fungi through enhancement the production of the defensive components such as, proteins and other defense mechanisms i.e., chitinase, layase, catalase and polyphenoloxidase [27–29]. It’s vital to remember that chitosan and Chitosan NPs effectiveness might change based on the pathogen type, plant species, application technique, and environmental circumstances, among other things.

Therefore, the best application techniques for certain crops and diseases, extensive research and field tests are required. The novel theoretic has been elevated that chitosan NPs can favorable influence on crop and soil microbial societies as AMF [30]. El Amerany et al. [31] reported that combined chitosan with AMF increase the stem cortex of tomato strains and improved sugar and protein content more than the non-inoculated one. The purpose of the current investigation was to allow protection of VF plant against *R. solani* root rot disease and enhance faba bean plant defenses to combat pathogens to limit losses by using bio-control agents. The effectiveness of chitosan in nano form as a bio agent in comparison to chitosan in its normal size and fungicides is also being investigated. Additionally, investigations are also being done into using Chitosan NPs and chitosan either individually or in combination with AMF and their potential effect on *R. solani* root rot to maintain a good production.

## Results

**Fig. 1:**
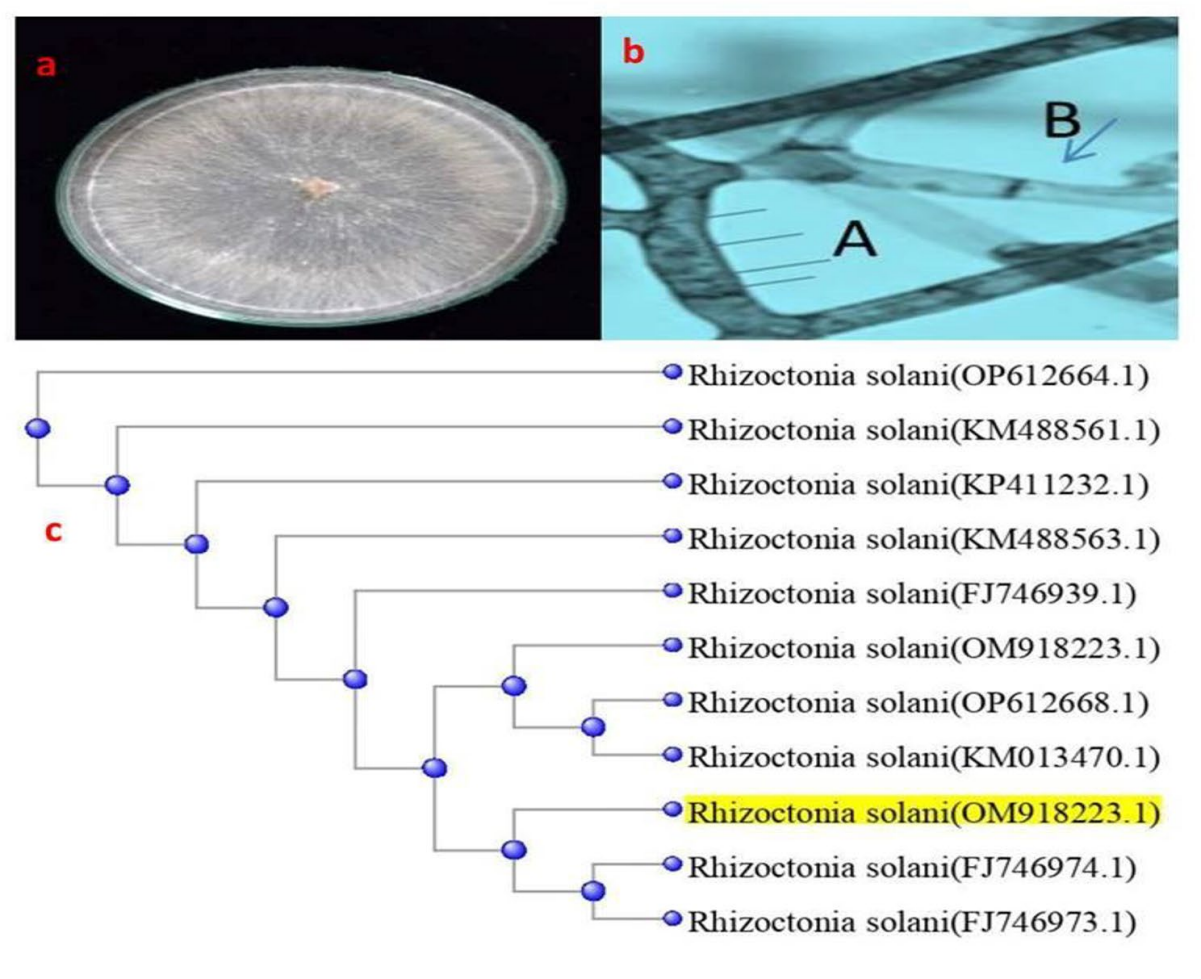
Phenotypically and molecular identification of *R. solani.***(a**): Growth of *R. solani* on PDA plate,**(b)**: *R. solani* examination under light microscope, A = Nuclei of *R. solani*, B = septa within hyphae. **(c)**: Multiple Sequence Alignment and phylogenetic tree of *Rhizoctonia solani* GenBank **(ITS: OM918223)**, showing its identity with the most similar *Rhizoctonia solani*, GenBank accession no.s, FJ746974, KM013470, FJ746973 and KM488561, respectively

Under the experimental conditions, it could be approved that the fungal species showing root rot disease belong to class; *Agaricomycetes*, represented by *R. solani*. Rhizoctonia isolates tested fitted well with the characters of *R. solani* as similar in number of nuclei, sclerotium production, brown pigmentation, constriction of the branch base of hyphae and formation of septa within a short distance of the origin of branches. The results showed the phenotypically characters of *R. solani* that grown on Potato dextrose agar (PDA) plate, Fig. (1a). From the examination test, isolated *R. solani* was polynucleate which containing 4 to 8 nuclei/apical compartment or moniloid hyphal cell, Fig. (1b). BLAST analysis showed that the ITS sequence of the tested fungal isolate in the present investigation is *R.solani* with accession number OM918223. From the phylogenetic analysis, the tested *R.solani* showed 99.86, 99.72, 99.72, and 99.72% identity with other *R. solani* isolates with GenBank accession nos., FJ746974, KM013470, FJ746973 and KM488561, respectively, Fig. (1c), and the sequence of the representative isolate was deposited in GenBank as (ITS: **OM918223**).

**Fig. 2:**
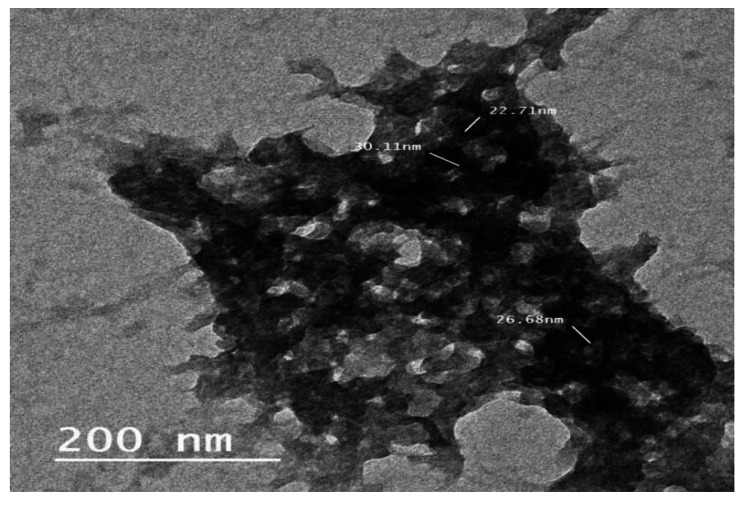
Characterization of Chitosan NPs: TEM of Chitosan NPs with spherical shaped

**Fig. 3:**
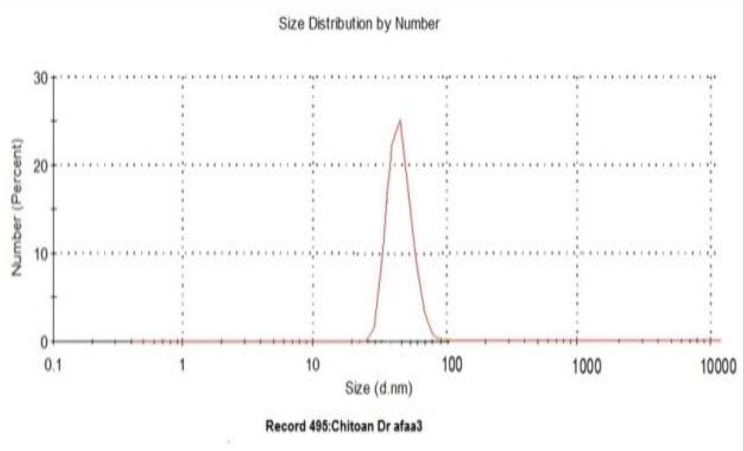
Characterization of Chitosan NPs: Dynamic light scattering (DLS) measurements of chitosan NPs with diameter at about 78.5 nm

**Fig. 4:**
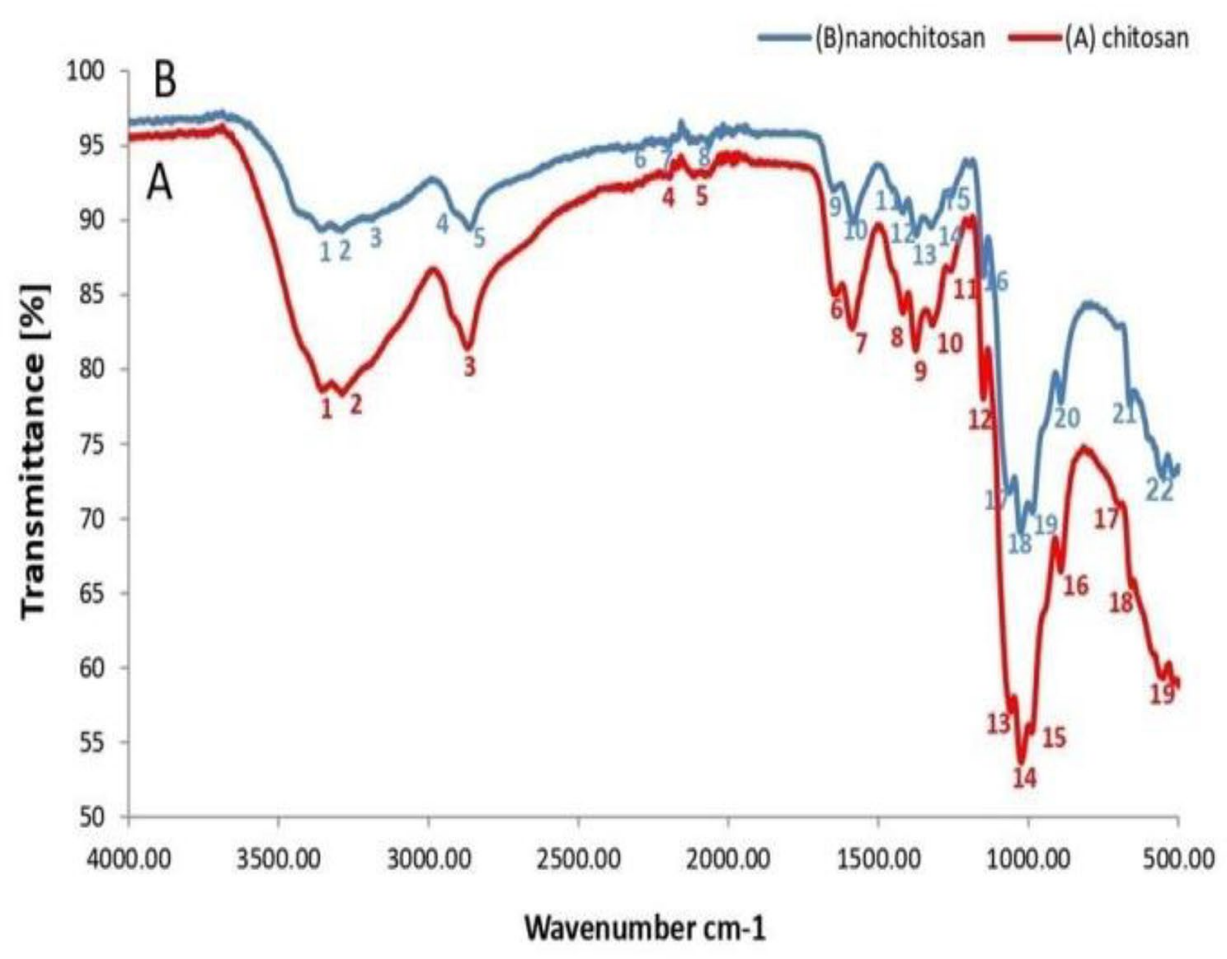
Characterization of Chitosan and Chitosan NPs: (A) FTIR of Chitosan, (B) FTIR of Chitosan NPs

The data appointed herein exhibited that the morphology for synthesized chitosan NPs were spherical shaped (Fig. 2) with diameter at about 78.5 nm (Fig. 3). To supplemental establish the existence of functional groups, Chitosan and Chitosan NPs were analyzed by FTIR (Fig. 4A,B) that exhibited the absorption bands at 3354,2288,2870,1646,1588,1418,1259,1150,656,581 and 418 cm^− 1^ which characterized the principle functional groups in chitosan, while the spectrum of chitosan NPs showed bands at 3428,3328,3289,2906,2861,1646, 1584,1418,1258,1150, 1026, 690, 593 and 448 cm^− 1^ which characterized the functional groups in Chitosan NPs. The difference in bands between chitosan and its nano-chitosan illustrated by the presence new band at 3428 cm^− 1^ in Chitosan NPs, and also the high frequency bands at 3289 and 2906 cm^− 1^. In addition to high frequency bands at 690, 593 and 448 cm^− 1^ indicated the change in Chitosan NPs spectrum. Whereas, the peaks at 3428 and 3354 cm^− 1^ are attributed to –NH_2_ and –OH groups stretching vibration. The peaks at 1646 cm^− 1^ and 1588 cm^− 1^ are assigned to the CONH_2_ and NH_2_ groups, respectively. These peaks shift into 1646 and 1584 cm^− 1^ in the FTIR spectra of Chitosan NPs which caused by the interaction between NH_3_ + groups of chitosan and phosphate groups of TPP. Further, the peak at 1026 cm^− 1^ which appears in the FTIR spectra of Chitosan NPs shows characteristic of P = O stretching vibration from phosphate groups.

**Fig. 5:**
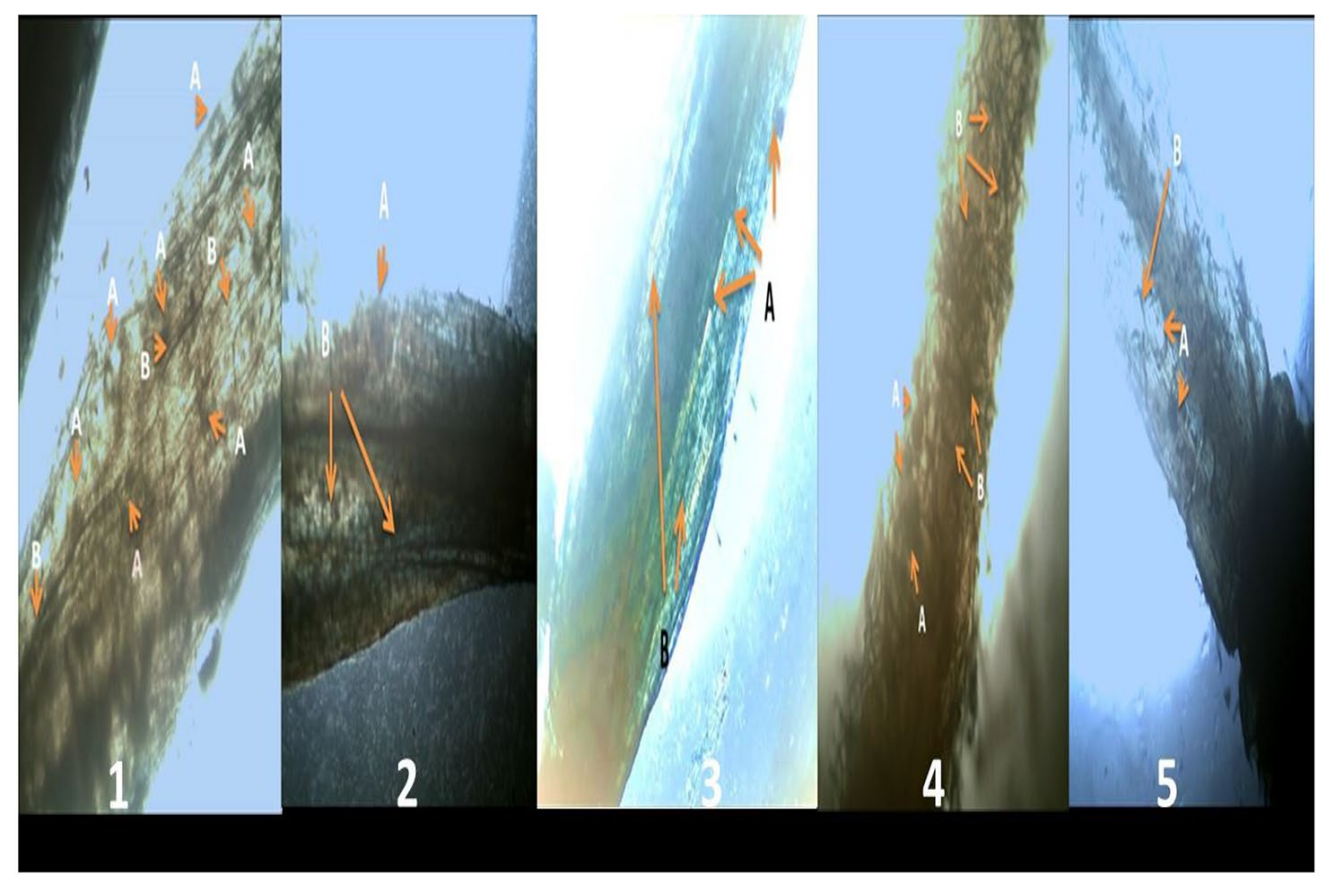
Colonization of (arbuscular vesicles and mycelium) of AMF in *Vicia faba* plants infected with *R. solani* (**A) = Vesicles; (B) = mycelium;(C) = Arbuscular. (1)** AMF + healthy plant, **(2)** AMF + infected plant with *R. solani*, **(3)** AMF + chitosan + infected plant, **(4)** AMF + Chitosan NPs + infected plant, **(5)** AMF + Fungicide + infected plant

Data given in Table (1) showed the *R. solani* preceded pre-emergence infection; post-emergence damping-off and root rot diseases with different percentages. *R. solani* caused about 77.8%; 66.6% of DS; DI, respectively. Therefore, *R. solani* was showed to be an aggressive pathogen and was used for further studies.

Considering to mycorrhizal infection frequency (F%) of VF plants; the results in Table (2) showed that there is no significantly difference between mycorrhizal infection of plants after 35 and 70 days of sowing( P.value ≥ 0.05). The most potent percentage of colonization by AMF was obtained in healthy plants at about (80%).In addition, F% increased through the increasing in VF age in most treatments after 35 to 70days. Where, (F%) after 35 days of infection of VF by *R. solani* were increased in the treatments of Chitosan NPs (80%) followed by chitosan (70%) compared with control (50%) but no change of F % in fungicide inoculation (50%). In addition, different morphological characteristics of AMF colonization as arbuscular, Vesicles and mycelium are showed in Fig. (5). Further, mycorrhizal colonization amplified and more obvious in the presence of Chitosan NPs than the other treatments as shown in Fig. (5).

From the results of Table (2), the dependency of VF plants on mycorrhizal association (MD%) varied with different treatments and these dependencies were higher after 70 days than that after 35 days. The maximum value of mycorrhizal dependency accounted for 23.3% and 48.2% after 35 and 70 days of plantation, respectively in AM plants infected with *R. solani*. It also notes that MD % of AM plants was decreased in presence of Chitosan NPs or chitosan.

The results in Table (2) revealed that the growth of VF plants represented by dry weight was affected by the different treatments used in this investigation. AMF improved the dry weight of the inoculated plants by about 16% and 40% after 35 and 70 days of plantation respectively. On contrary, AMF increased the growth of VF inoculated with *R. solani* by about 23.8% after 35 days and 48% after 70 days of sowing. The results also showed the percentage of improvement of the dry weight of VF plants decreased into 11.4% and 28.4% after 35 and 70 days of plantation, respectively in plants co-inoculated with AMF in the presence of Chitosan NPs. While in the presence of micro chitosan, the percentage of increasing in dry weight of AM faba plants accounted 20% after 35 days of sowing and 31.5% after 70 days of sowing.

The data of Table (2) presented that the disease incidence in VF plants was appeared to correlate with the presence and absence of AMF. The incidence of root rot symptoms issued using *R. solani* appeared in 80% and 83.4% after 35 and 70 days of plantation, respectively in non-mycorrhized VF plants. While in AM plants, the extent of root rot symptoms was constricted to 64.2% and 51.2% after 35 and 70 days of plantation, respectively. The presence of Chitosan NPs in diseased plants decreases the incidence of the disease either in the absence or with AMF and accounts for 43.6% and 23.8%, respectively after 35 days of plantation. After 70 days of sowing, the incidence of the disease accounted for 29.3% in absence of AMF and 10% with AMF. The subsistence of chitosan also affects the incidence of the disease accounting for 52.5% and 33.4% in absence of AMF after 35 and 70 days of plantation, respectively. On the other hand, the disease incidence in the presence of AMF was accounted for 33.9% and 25% after 35 and 70 days of sowing. In addition, the presence of fungicide effects on the incidence of the disease accounting for 53.9% and 33.4% in absence of AMF after 35 and 70 days of plantation, respectively. In addition, the disease incidence in the presence of AMF was accounted for 43.8% and 29.1% after 35 and 70 days of sowing. In this connection, the disease severity of VF plants was appeared to be correlated with the presence and absence of AMF. Where, the presence of AMF with VF plants exhibited decreasing in disease severity percentages of VF plants infected by *R. solani*. In addition, disease severity percentages of VF plants infected by *R. solani* were significantly decreased in the presence of Chitosan NPs compared to chemical one by fungicide as shown in Table (2).

The induction effect of the treatments used in VF inoculated with *R. solani* on the concentration of nitrogen, phosphorous, and potassium is tabulated in Table (3). The presence of pathogen causes the reduction of N, P, and K concentration in plant tissue while the suppression degree was significantly minimized in mycorrhizal plants at variance in non-mycorrhizal ones. Nitrogen, phosphorus and potassium concentrations in plant tissue were significantly more in mycorrhizal plants than that of non-mycorrhizal ones either with or without the pathogen. In addition, these elements were sufficiently enhanced with increases in the age of the plant. The data introduced in Table (3) also presented the improvement of nutritional status of diseased VF plants with chitosan NPs either with or without AMF but the degree of improvement was more considerable in mycorrhizal plants than that of non-mycorrhizal ones. In AM plants, the nitrogen was increased by about 40% and 47% after 35 and 70 days of plantation, respectively compared with diseased AM plants in absence of Chitosan NPs. Phosphorus contents in AM plants were improved by about 52% and 65% after 35 and 70 days of plantation respectively compared with diseased AM plants in absence of Chitosan NPs. On the other hand, Potassium did not exhibit significant improvement in presence of Chitosan NPs. The presence of chitosan or fungicide in AM plants also improves the nutritional status of diseased plants but in lower values than that obtained in case of Chitosan NPs with AM ones. The percentage of improvements of nitrogen element was accounted for 38% and 35% after 35 and 70 days of plantation, compared with diseased AM plants in absence of chitosan. The improvement of Phosphorus rate accounted for 20% and 27% after 35 and 70 days of plantation, respectively compared with diseased AM plants in absence of chitosan. On the other hand, in the case of Potassium, the improvement occurs only after 70 days of sowing and accounts for about 23% in comparison with diseased AM plants in absence of chitosan.

The improvement of VF plant growth infected by *R. solani* using AMF with Chitosan NPs or chitosan is shown in Table (4). The percentage of total soluble sugar was decreased in the presence of the pathogen either in mycorrhized or non-mycorrhized ones. It was decreased from 18.76 to 15.95% and from 23.63 to 20.8% after 35 and 70 days of plantation, respectively in non-AM plants. While in AM plants, it decreased from 21.87 to 19.17% and from 24.8 to 22.22% after 35 and 70 days of plantation, respectively. The coincide effect of the presence of Chitosan NPs and AMF leads to an increase in the % of Total soluble sugar (T.S.S.) of diseased VF plants from 19.17 to 22.21% and from 22.22 to 25.0% after 35 and 70 days of plantation, respectively. On contrary, the presence of chitosan with AMF causes a slight increase in % of T.S.S. of diseased VF plants especially after 70 days accounting for 22.22 to 23.05%.

From the results given in Table (4), it could be noticed that the whole chlorophyll contents excessed relatively among different treatments compared with infected plants, and also showed that the increase in mycorrhizal ones is considerable than given in non-mycorrhizal ones. In addition, the chlorophyll content was significantly excessed by proceeding the age of the plant and gave the highest content in mycorrhizal plants inoculated with chitosan NPs. The presence of Chitosan NPs with AMF increased the total chlorophyll by 27% compared to diseased AM plants after 35 days of sowing while, after 70 days the rate of increases accounted for about 23%. On contrary, the presence of chitosan with AMF causes increases of total chlorophyll in VF plants by about 17% and 12.7% after 35 and 70 days of plantation, respectively.

The effects of different treatments in phenol rate and protecting enzymes activities in mycorrhizal and non-mycorrhizal faba bean infected by *R. solani* are presented in Table (5). Phenol content was considerably increased in mycorrhizal and non-mycorrhizal plants due to pathogen infection. In addition, all processing considerably amplified phenol content compared with healthy plants and still were more considerable in AM plants than non-AM ones. The results of the Table (5) mentioned that the total phenol rate in VF inoculated with AMF increased in healthy plants by about 1.5 and 1.8 fold after 35 and 70 days of plantation, respectively more than that of non-inoculated. On the other hand, the total phenol rate in VF inoculated with AMF increased in diseased plants by about 2 and 2.4 fold after 35 and 70 days of plantation, respectively more than that of non-inoculated. Moreover, the inoculation of Chitosan NPs into infective AM plants exceeded the total phenol content at about 2 and 2.2 fold after 35 and 70 days of plantation, respectively more than that of non-inoculated. In addition, the total phenol rate in VF inoculated with AMF increased in chitosan or fungicide treatment by about 2 fold after 70 days of plantation more than that of non-inoculated.

The results of Table (5) clearly showed that the defense enzymes’ activities were significantly increased under the treatments used when compared with infected and healthy plants that still higher in mycorrhized plants than that on non-mycorrhized ones with various degrees ranging from 7 to 69% for POX enzyme and 16–50% for PPO enzyme. The results also showed that the maximum activity of POX enzyme was observed in the case of AM diseased plants after 35 and 70 days accounting for 14.05 and 11.38 ∆ absorbance unit min^− 1^ g FW^− 1^, respectively. The presence of Chitosan NPs with Faba plants inoculated by AMF comes to be in the second rank in POX activity accounting for 13.9 and 10.65 ∆ absorbance unit min^− 1^ g FW^− 1^. While, the addition of chitosan to AM plants activates POX enzymes to a lesser extent than that in the presence of Chitosan NPs with AM plants accounting for 13.1 and 10.59∆ absorbance unit min^− 1^ g FW^− 1^ after 35 and 70 days of plantation, respectively.

With respect to, PPO enzyme activates shown in Table (5), the maximum activities were observed in AM plants infected with *R. solani* accounting for 6.79 and 5.29 ∆ absorbance unit min^− 1^ g FW^− 1^. While, in the presence of Chitosan NPs with AM plants the PPO activities were accounting for 5.56 and 4.92 ∆absorbance unit min^− 1^ g FW^− 1^ after 35 and 70 days of plantation, respectively. On contrary, PPO activities in the case of chitosan with AM plants accounting for 5.67 and 4.14 ∆ absorbance unit min^− 1^ g FW^− 1^ .

**Fig. 6:**
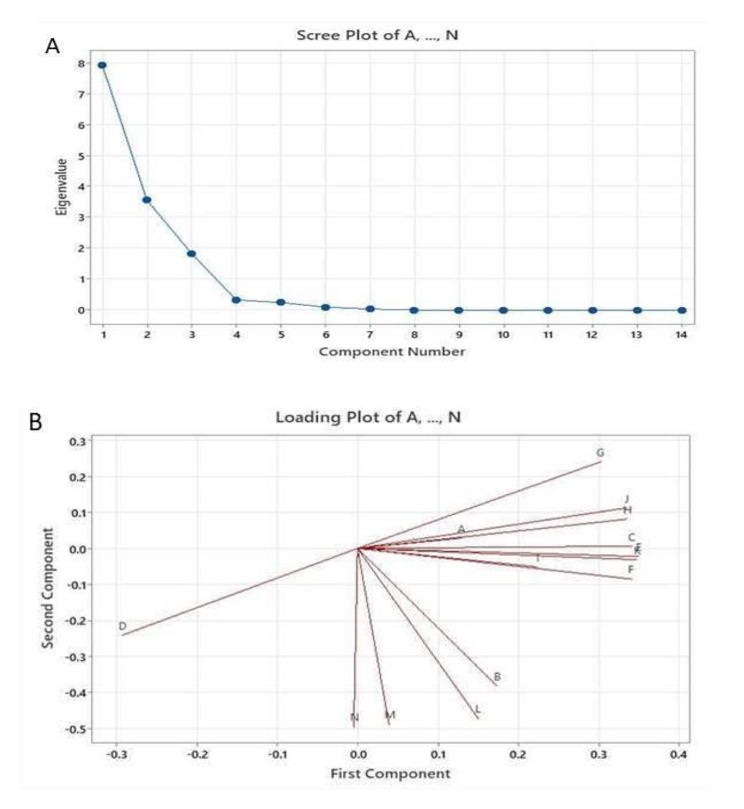
The scree plot of eigenvalue (A) and loadings plot (B) for the component number of the measured parameters for Faba bean plants under infection with *R.solani* in response to treatments with AMF and Chitosan NPs: A: F% Frequency of mycorrhizal root segments, B: Root / Shoot length, C: Mycorrhizal dependency (MD) D: Dry Wt (g), E: Disease incidence % F: Nitrogen content, G: Phosphorus content (P), H: K content, I: TTS%, J: Chl.a, K: Total chloro, L: Phenols content, M: Peroxidase POX activity, N: Polyphenoloxidase activity

Principal component analysis (PCA) was carried out regarding the parameters that measured on VF plant infected with *R. solani* and treated by either AMF or Chitosan NPs (Fig. 6; A&B). The first three measured components F%, R/S%, and MD% with greater eigenvalues than the others. Where, the mycorrhizal colonization frequency (F%) was described of about 80%. Accordingly, PCA analysis that gave evidence to the importance of parameters especially (F%); as it was considered a good and more reliable indicator for development of AMF in the presence and absence of Chitosan NPs under the presence of *R. solani*.

## Discussion

Mycorrhizal association is an assistance to plants for management the adverse ecological conditions [17]. In the present results, *R. solani* has the ability to cause root rot and damping-off symptoms for VF and disease incidence in VF plants under different treatments ranged from 23.8 to 80% and 10 to 83.4% after 35 and 70 days of sowing, respectively. Deleterious influence of fungus *R. solani* on morphological characteristics of VF may be caused by deterioration of roots which minimize assimilation for substantial minerals and water [2, 32, 33]. On the other hand, the whole parameters of growth of VF plants grown under the presence of *R. solani* enhanced over with AMF and Chitosan NPs.

The data proved that using AMF correlated by Chitosan NPs may assist in inhibition the deleterious influence of pathogenic fungi. As reported previously, AMF and nanoparticles has the ability to enhance the resistance of adverse plants to different pathogenic fungi [17, 34].

The results employed herein have showed that the AMF infection frequency (F%) in diseased plants was increased from 50% to 80% in the presence of chitosan NPs at variance without chitosan NPs. Since, AMF and nanoparticles plays important role in plant disease control [17]. Moreover, the positive influence of Mycorrhizal colonization on plants growth parameters activation might be due to the physiological enhancement within the plant by the effect of the symbioses mycorrahiza as gibberellins increasing [17, 35]. Further, the data proved that Chitosan NPs is the most positive effective on the dry weight in AM faba plants as obtained by El-Amerany et al. [31] who reported, the formula of chitosan and AMF enhanced the cortex of the stem that is responsible for minerals distribution in tomato plants.

In the present investigation, the results exhibited that the incidence of the disease was much lower in VF treated with AMF compared to non-treated one. Moreover, the presence of Chitosan NPs with AM plants reduced the incidence into the lowest value; it was decreased into 23% and 10% of plants, respectively. These results are in agreement with those of Aljawasim et al. [36] who reported that inoculation with AMF reduced the disease severity caused by *R. solani* in cucumber. Moreover, the study indicated that treatment of faba bean plants with chitosan or Chitosan NPs reduced DS% and DI caused by *R. solani* due to their potential effect on the pathogens and on the plants. These results are similar with Hassan and Chang [26] who observed the antimicrobial impact of chitosan or Chitosan NPs in agricultural field experiments which Chitosan decreases H+-ATPase that accumulates inside the cell due to rapid potassium outflow causing changes in H+/K + exchange transport across the membrane of *Rhizopus stolonifera*. Moreover, similar observed by El Amerany et al. [31]. Chitosan and Chitosan NPs can stimulate the plant’s natural defense mechanisms, triggering an enhanced immune response. This includes the activation of defense-related genes, the production of antimicrobial compounds, and the reinforcement of the plant’s cell walls enhancing the plant resistance. They can be used as coatings, films, or sprays to protect plant surfaces from pathogen attack. By forming a physical barrier, they prevent pathogens from entering plant tissues and causing infections. As a result, plants become more resistant to pathogens, reducing the severity of diseases. On the other hand, they can inhibit the growth and development of pathogens by disrupting their cell walls, interfering with their enzyme systems, and inhibiting their replication. These results confirmed the synergistic effects of Chitosan NPs with AMF for induction of the resistance of faba plants to root rot disease [28].

In the present investigation, there is a difference in the dependency of faba plants on AMF with different proportions between the tested treatments. Whereas, AMF improved the plant growth, some nutrients obtained, sugar and chlorophyll content than non-mycorrhizal plants, it can mitigate the unfavorable influence of pathogenic and increased the plant resistance to root rot disease. These results are agreement with previous studies [37], which indicated that AMF got better plant growth and play an important role in disease protection. As well as the mycorrhizal dependencies of faba plants were increased with the age of plants in all treatments as was reported by El-Gazzar et al. [17]. Similar results were obtained by El Amerany et al. [31] who found that, chitosan and AMF together and 10% compost improved the physiological and growth parameters. Moreover, the mycorrhizal dependencies of faba plants were increased with the age of plants in all treatments as mentioned by El-Gazzar et al. [17]. It is remarkable to observe the mycorrhizal dependency in the presence of Chitosan NPs were decreased to the lowest value (11.4% and 24.8% ) after 35 and 70 days of sowing; this indicating on the AM plants were less dependent on AMF. Although mycorrhizal dependency was low with AMF fused to Chitosan NPs, a high dry weight and the disease incidence showed maximum and minimum values all over the periods of planting. From the knowledge that; mycorrhizal dependency of plants is increasing under plant stresses. It could be conclude that the Chitosan NPs alleviates the negative stress of *R. solani* in AM faba plants and may interpret the beneficial effects of AMF and Chitosan NPs in both presences together [17] Thus, chitosan or Chitosan NPs were demonstrated as control products and plant protection against *R. solani*; this effect due to that chitosan inhibited fungal growth and development of *R. solani* [38]. The cationic nature of chitosan due to the subsistence of different amine signs that is the basic of their efficiency versus [17, 38, 39].

Close examination of the present data, nitrogen, phosphorus and potassium concentrations in plant tissue were remarkable potent in mycorrhizal plants than that of nonmycorrhizal plants either in the presence or without the pathogen [30]. Whereas, the presence of Chitosan NPs in AM plants leads to increasing nutrient content of plants by about 40% and 47% for nitrogen and 52% and 65% for phosphorus. So, the data of this study confirmed the synergistic effect between the presence of Chitosan NPs and AMF for improvement of nutritional status of diseased faba plants as reported in the previous studies [30, 31].

The chlorophyll content is one of the most important parameters reflecting the plant healthy. Whereas, there was a favorable relevance with chlorophyll content and the plant leaves and net assimilation photosynthesis of carbohydrate content. Carbohydrate comprises structurally polysaccharides cell wall such as pectin that acts as an important barrier versus disease invasion [31, 40]. In this connection, all treatments in mycorrhizal plants increased T.T.S, and chlorophyll contents more than non-mycorrhizal plants, and Chitosan NPs was more effective than chitosan compared to hygienic plants and infective ones. Also, the results were coincided that chitosan NPs and AMF leads to an increasing in the % of T.S.S. of diseased faba plants from 19.17 to 21.03% and from 22.22 to 25.0% and for chlorophyll content by 27% and 23% after 35 and 70 days of planation.

The present study demonstrated that AMF colonization enhanced phenolic rate in VF plants contracted by *R. solani* due to pathogen infection and Chitosan NPs more effective than other treatments. Previous data summarized by Rashad et al. [41] who indicated that AMF induced phenolic content in common been infected by *Fusarium*. Chandra et al. [42] mentioned that chitosan and Chitosan NPs induced the evaluation of the levels of total phenolic in plant around 20–24% over the control, in Chitosan NPs 3.5% higher than that of chitosan. Also, Lamba et al. [43] showed that the role of phenols as antioxidant, photoreceptor and antimicrobial due to its assignment as originator physical barrier, biosynthesis of specific phytoalexins and induce the structural barriers. In this connection, the beneficial effect of mycorrhizal colonization and chitosan NPs against root rot pathogens may be due to enhancing chlorophyll content, increasing total phenol and inducing the antioxidant efficiency as Polyphenoloxidase (PPO). Peroxidase and Polyphenoloxidase enzymes are playing a defensive role against invading pathogens, hence, the oxidation-reduction enzymes share with lignification in host plant cells during the resistance against the pathogens. Whereas, the present results showed that the defense enzymes’ activities were significantly increased under of the treatments used when compared with infected and healthy plants and still these efficiency were major in mycorrhized plants than that obtained in non-mycorrhized ones. These results agree with Rashad et al. [41] who indicated that, AMF increased induction PPO and POX activity in common been infected by *Rhizoctonia solani* and *F. oxysporum*. Furthermore, the results of this investigation demonstrated that Chitosan and Chitosan NPs have induction impacts in enzymes activity (POX and PPO) compared with healthy plants but less induction impact in the infected plants. This agree with that reported by Chandra et al. [41, 44]. In addition, the role of PPO in plant defense may be due to the virulent of polyphenol oxidase- created quinones (antimicrobial compound) to pathogen and alleviation applicable cellarer protein with quinones or phenolic composition as a substantial partition to pathogen infestation [45].

With a comprehensive look of the results of this investigation, it was demonstrated that the addition of AMF with Chitosan or Chitosan NPs led to a reduction in the incidence of infection of VF plants with *R. solani*, where the infection decreased from 80% in the diseased plant to 23% within AMF and Chitosan NPs and 33% within AMF and Chitosan after 35 days of sowing. While after 70 days of sowing, the disease reduced from 83 to 10% and 25% in Chitosan NPs and Chitosan, respectively with AMF. Although the deleterious effect of Chitosan on pathogenic fungi, does not impact mycorrhizal fungi [24, 26]. Chitosan had induced beneficial mycorrhizal activity; this was observed by the positive influence of chitosan with AMF in the reduction of the disease and improving the health and vitality of infected plants due to Chitosan-Mycorrhiza synergistic effect [46 ]. Furthermore, [46, 47] shown that there are interaction, of various nanoparticles and AMF which effect on its growth with neutral, positive and negative effects according to the NPs sort, diameter, dose and soil characters. Finally, all the previous results in the present study can be proved that the AMF colonization and Chitosan NPs increased VF plant resistance against the infection of root rot pathogens (*R. solani*). Chitosan NPs are more effective and powerful at fighting pathogens when fused by AMF.

## Conclusions

In the current investigation, the growth of faba bean root rot pathogen can be controlled using chitosan, Chitosan NPs and AMF as bio control with a high inhibition rate. Chitosan and Chitosan NPs are ecofriendly, biodegradable and anti- *R. solani* compared with fungicide. Moreover, the use of AMF and chitosan derivatives either in normal or in nano form induces the VF plant to resist root rot disease. Worth mention, the use of both bio-fertilizers Chitosan NPs and AMF has proven to be very beneficial for the growth of faba bean plants and induced plant resistance. Moreover, in our study, the application of Chitosan NPs and AMF together on plants grown promotes changes in all parameters studied more than using severally and more than other treatments. Hence, there is remarkable improvement and an increase in root and shoot growth, chlorophyll fluorescence, nutrient content and defense enzymes activities, Moreover, their superior ability to suppress *R. solani* root rot disease in faba bean plants by enhancing their anti-fungal activity against *R. solani*. From the previous results, we recommend using Chitosan NPs and AMF together as bio-control agents to overcome the deleterious effects of *R. solani* root rot and enhance growth and physiological activities of the fab bean plant.

## Materials and methods

### Source of mature seeds

*Vicia faba* L. seeds (Giza 843) were brought by Crops Department, Agric. Res. Center (ARC), Giza, Egypt. They were sanitized using 0.01% HgCl_2_, purified using distilled H_2_O and introduced in pots with soil about 2000 g [48].

### Fungicide and Rhizobium leguminosarum inocula

Fungicide; Rizolex T.50 wp. (Tolclofos-methyl) was gotten from Al-Gomhoria Company, Egypt, applied as a coating for seeds at 3 g/kg seeds according to the recommended dose by the Ministry of Agriculture.

*Rhizobium leguminosarum* biovar viciae had been kindly provided from the Bio-fertilizer production unit, Soil, Water and Environment Research Institute, Egypt; this was used for soil inoculation before sowing. All legume species requires specific rhizobium strain due to existence of symbiotic relationship between them and faba bean increasing the chance of successful nodulation and nitrogen fixation. Thus, improving soil fertility, reducing costs and minimizing impact on the environment at the same time improving the productivity [49].

### Mycorrhizal inocula and potting mixture

A mixture of AMF was brought by Plant Pathology Research Institute, Agric. Res. Center (ARC) Giza, Egypt. AMF spores were in suspension at 1 × 10^6^ unit/L from *Funniliformis mosseae* and *Gigaspora gigantean*. The mixture of mycorrhiza was reactivated by trapping with surface-sterilized Sudan grass seeds (*Sorghum vulgare*) using 1% sodium hypochlorite then rinsed them with sterile distilled H_2_O, Sudan grass seeds were cultivated in sterilized pots (30 cm diameter) including autoclaved sandy loam soil (1: 1 v/v), after inoculation with AMF, then irrigated gently. These cultures were grown for two successive cycles (four months) under controlled conditions in day/night of 16/8 period, 20–25 °C and 50% humidity, then Sudan grass were separated from the surface of soil ; the soil was used as AMF inocula [17].

### Isolation of R.solani and its molecular identification

The experimental pathogenic fungus was isolated and purified from infected VF plants infected with root rot disease, provided from Al-Dakahlia region, Egypt, (90Km north Cairo). The infected root parts of VF were collected, thoroughly washed by tap water and cut using a sterile scalpel, then surface sterilized by 20% NaOCl for 5 min, the sterilized pieces were rewashed twice with sterilized water for 2 min, then cut into small pieces and dried; then were cultivated potato dextrose agar (PDA, Oxoid) supplied by the antibiotic streptomycin - sulfate 100 µg/ mL). After incubation of the PDA agar plates for 6 days at 25^0^ C, the growing fungus was purified on the same medium [50] and was identified morphologically [51, 52]. *R. solani* was identified according to phenotypically characteristics througth colony color (pigmentation), growth pattern and sclerotial formation pattern.The nuclei was stained by transfering *R. solani* isolate that was grown on PDA media on glass slide and covered by water agar. Growing hyphae were stained by trypan blue to examine the nuclei content especially in the apical part of fungal hyphae [51]. Stained hyphae were examined under light microscopy (400X) to count the number of nuclei/apical compartment then photographed.

### DNA extraction of the tested Pathogen

DNA extraction was isolated from mycelia of *R. solani* 14-day; old cultures using Lee and Taylor method [53]. The pellets of DNA were washed by ethanol, solubilized in dist. H_2_O, and kept at -20 °C.

### PCR conditions, sequencing and multiple sequence alignment

According to White et al., [54], the ITS part of rDNA was amplified with ITS 1 (5′ TCC GTA GGT GAA CCT GCGG 3′) and ITS 4 (5′TCC TCC GCT TAT TGA TATGC 3′) primers. Thermo-cycler program for amplification of the ITS region was: 95 °C about 3 min then by 40 turning of 95 °C about 30s, 68 °C about 45 s, 72 °C about 90 s. The last reaction was conducted at 72 °C for 8 min.

DNA sequences were exhibited by the amplified PCR sequencing output through the ABI Prism 3130xl Genetic analyzer, in both directions with the same primers ITS 1 that were mentioned above [54], sequencing were implemented in (Macrogen Corp., Korea). DNA sequences have been stabilized in the Gene Bank NCBI. Multiple Sequence Alignment by CLUSTALW, phylogenetic tree were made with the function “build” of ETE3 v3.1.1 [55].

### Synthesis of chitosan nano particles

Chitosan was obtained from Chitosan Egypt Company (Giza, Egypt). The ionic gelatin method was used for preparation of chitosan NPs [56–58]. Briefly, chitosan was dissolved to create a 0.5 mg/mL chitosan soln in an aqueous solution of acetic acid. Acetic acid content was 0.4 fold than (0.2 mg/mL) chitosan concentration. Using a magnet stirrer, the chitosan soln was stirred all night long at room temperature. The final solution had a pH of about 3.6, which was changed to 4.7–4.8 by adding 20 wt % of an aqueous sodium hydroxide solution. To get rid of any remaining traces of insoluble particles, the chitosan solution was subsequently filter-sterilized using Millipore filters (0.25 μm, Amicon, Mumbai, India). Sodium tripolyphosphate solution (TPP), 0.5 mg/mL was settled in deionized water and then sterilized using a Millipore filter (0.25 μm). Then, 3 mL of 2–4 °C TPP was dropped into the chitosan solution after heating 10 mL of chitosan solution in a beaker within a water bath at 60 °C for 10 min. After 10 min., the suspension that obtained was examined further.

### Instrumental analysis used for characterization of chitosan nano particles

#### Transmission electron microscope (TEM)

The shape and morphology of chitosan NPs measured with TEM model JEM-1230, Japan, operated at 120 kV, through the highest resolution 600 × 10^3^ for 0.2 nm [59].

#### Dynamic light scattering system (DLS)

The DLS was typically used to determine the hydrodynamic diameter of materials by analyzing the diameter of particles [17]. DLS (Malvern, UK) research was conducted at the Agriculture Research Centre (ARC), Giza, Egypt’s Regional Center for Food and Feed.

#### Fourier Transform Infrared Spectroscopy (FTIR)

Amino acids residues were discovered in Chitosan and Chitosan-NPs using FTIR (Thermo Nicolet model 6700 spectrum); Located in Cairo University’s Micro-analytical Center in Giza, Egypt [17].

#### Pathogen inoculum preparation and pathogenicity test

A series of sterilized conical flasks (250 mL) including 200 mL PD broth medium were inoculated by actively growing *R. solani* (2 × 10^5^ propagules/ mL) obtained from the peripheral of 7 days old colony. The inoculated flasks were then incubated for 5 days at 25^0^ C.

To study the infection ability of VF root by *R solani*, eighteen pots (30 Cm diameter) were prepared and sterilized by formalin solution (5%) for 15 min. Then, they were left for 1 week for evaporation of formalin in open air. Each pot containing its soil was infected with fungal propagules suspension (5mL /Kg soil) by thoroughly mixed with soil according to Manashi and Dutta [60]. The pots were wetted daily for 7 days to allow growth of the experimental fungus. Sterilized VF seeds were planted (5 seeds/ pot).Nine pots (5plants/ 1pot) for each experiment either inoculated or non-inoculated were used and assessed in each experiment. Three replicates of inoculated or un-inoculated pots (control) were kept in greenhouse at controlled condition (19^0^ C night, 25^0^ C days and 12 h photo period) then were irrigated when necessary. The disease assessment was measured with the rate of pre-emergence damping-off after fifteen days of plantation. Post-emergence was performed at 30 days after sowing and survival of plants was performed after 60 days of planting as f: Pre-emergence damping-off % = the number of non-germinated seeds / total number of sown seeds × 100; Post-emergence damping-off % = the number of dead seedlings / total number of seeds sown × 100; Survived plants % = No. of survived plants / total sown seeds number × 100. Disease severity % (DS) was determined according to Rashad and AL-Askar [61] through a 0–5-parameter range: 0- no symptoms, 1.slight lesions, 2.lesion coalescing surround the root, 3.lesion diffusing into the root and root tips that being influenced, 4. root parts that full influenced and only a slight amount of white, uninfected tissue left, 5.completely infected root. Then the severity of root rot was calculated using the following equation.

DS = Σ ab / AK x100

Where: Ds = Disease Severity, a = no. of the disease plants have infection.

symptoms, b = infection degree, A = plant total number, K = the highest infection.

Moreover, disease incidence(DI%) = no of infection plants / total no of examination.

plants x 100.

### Effect of arbuscular mycorrhiza, chitosan and synthesized chitosan nanoparticles against root rot pathogen

A series of pots (30 Cm diameter); each containing 3.5 Kg sterile soil (2:1 V/V) clay: sand were prepared. The experiment was carried in 3 replicates for each treatment. The treatments are given in Table (6). Eighteen pots for each treatment and each treatment planted with VF seeds cv. Giza 843.The first treatment was used as healthy control without any infection. While, the other treatments infected by *R.solani*. The third and fourth treatments were prepared through soaking VF seeds for 10 h. in 1000 ppm of chitosan and chitosan nanoparticles that synthesized mentioned above. While, the fungicide (Rizolex) was used as seed coating (3 g/ Kg seeds) before planting for preparation of fifth treatment. All treatments were inoculated with *Rhizobium leguminosarum* biovar viciae as a bio-fertilizer. Seeds were planted at the rate of 5 seed/ pots. Treatments and control were allowed for growth of VF for 70 days in a growth chamber at 25/20˚C day/night, 11 h day, with 60–70% of relative humidity and then were irrigated when necessary [61]. The plants were harvested collected at 70 days after sowing date. Observations were measured at 35 and 70 days of inoculation. In addition a pathogenicity test as disease indices (DI) and disease severity (DS) were assessed on *R. solani* for all treatments at 35 and 70 days of inoculation.

**Table 1:** Pathogenicity test of *R.solani* on *Vicia faba* plant

Treatment	Total no of examined plants in all pot (A)	Pre- emergence% (Dead seed)	Post emergence% (Dead seed)	Survival %	Disease Incidence (DI)%	Disease severity% (DS)
Control	**45**	**0b**	**0b**	**100a**	**0b**	**0b**
*R.solani*	**45**	**1.7a**	**3.4a**	**46.7b**	**66.6a**	**77.8a**

LSD: At significant level (P *>* 0.05).Sample symbols (a.a) mean non significant difference (a.b) mean significant difference

**Table 2:** AMF colonization, root / shoot ratio, Mycorrhizal dependency %, Dry weight and Disease Incidence of *Vicia faba* plants under different treatments

		F %		R/ S		M D %		DW	D I%		DS%	F %		R/ S		MD%	DW DI%			DS%
		After 35 days of sowing										After 70 days of sowing								
Healthy Plants	NM	-	42%		-		0.75b			-	-	-	18.2%		-			11.287b	-	-
	M	80	43%		16.9		0.877a			-	-	80	21.3		40.2			15.833a	-	-
Diseased Plants	NM	-	41.4%		-		0.62c			80a	70a	-	17.3%		-			7..023c	83.4a	75a
	M	50	42%		23.3		0.768b			64.2ab	65ab	50	21%		48.2			10.414b	51.2ab	51.70ab
Diseased plants+ Chitosan NPs	NM	-	45%		-		0.793b			43.6b	15.80e	-	19.6%		-			11.413b	29.3bcd	11.70de
	M	80	45%		11.4		0.884a			23.8cd	9.10ef	80	27%		28.4			14.657a	10ef	8.30e
Diseased plants+ Chitosan	NM	-	44%		-		0.717b			52.5c	27.50b	-	19.2%		-			10.15b	33.4bc	25b
	M	70	45%		14.3		0.82a			33.9e	21.70bc	80	25.9%		31.5			13.353a	25d	17.50c
Diseased plant + Fungicide	NM	-	42%		-		0.715b			53.9	18.40cd	-	19.4%		-			10.124b	33.4bcd	14.20cd
	M	70	43%		16		0.782a			43.8	15e 70		22%		33.9			13.12a	29.1e 12.5d	

F% Frequency of mycorrhizal root segments, NM: Non-mycorrhizal plants and M: Mycorrhizal plants, DW: Dry weight MD% mycorrhizal dependency = [(DW of M - DW of NM)/ DW of M] x 100, DI%: Disease Incidence; R/S: Root / shoot. LSD: At significant level (P *>* 0.05).Sample symbols (a.a) mean non significant difference, (a.b) mean significant difference

**Table 3:** The Effect of mycorrhizal infection with different treatments on nutritional elements (N, P, K) in *Vicia faba* under infection with *R. solani*

		After 35 days of sowing			After 70 days of sowing		
		N	P	K	N	P	K
Healthy Plants	N M	2.81b	0.27bc	2.54 cd	4.17bc	0.404d	4.037bc
	M	3.19a	0.33a	2.86a	5.14ab	0.530ab	4.16abc
Diseased Plants	N M	1.96e	0.205d	2.54 cd	2.98d	0.303e	2.81e
	M	2.39 cd	0.256d	2.86a	3.87c	0.403 cd	3.23d
Diseased plants+ Chitosan NPs	NM	2.42 cd	0.303abc	2.30 fg	4.42b	0.456c	2.81e
	M	3.32a	0.39a	2.77b	5.7a	0.665a	3.23d
Diseased plants+ Chitosan	N M	2.21de	0.247 cd	1.99 h	3.933c	0.402d	3.87c
	M	3.31a	0.309ab	2.44de	5.243a	0.513b	4.233abc
Diseased plant + Fungicide	NM	2.23de	0.245 cd	1.91 h	3.936c	0.402d	3.97c
	M	3.31a	0.307ab	2.44de	5.271a	0.511b	4.237abc

LSD: At significant level (P *>* 0.05).Sample symbols (a.a) mean non- significant difference (a.b) mean significant difference. NM; Non-mycorrhizal plants and M; Mycorrhizal plants. N: Nitrogen; P: Phosphorus, K: Potassium

**Table 4:** The Effect of mycorrhizal infection with different treatments on total chlorophyll contents and % of total soluble sugar in *Vicia faba* infected with *R. solani*

	After 35 days of sowing					After 70 days of sowing			
		% Of total soluble sugar	Chl. a (mg/g Fresh weight)	Chl. b (mg/g Fresh weight)	Total Chlorophyll (mg/g Fresh weight)	total soluble sugar	Chl. a (mg/g Fresh weight)	Chl. b (mg/g Fresh weight)	Total Chlorophyll (mg/g Fresh weight)
Healthy Plants	N M	18.76ef	2.233abc	1.75c	3.981c	23.63bc	2.5 cd	1.877ab	4.377c
	M	21.87ab	2.082bc	1.502ab	3.584b	24.80a	2.338abc	1.908ab	4.69ab
Diseased plants	N M	15.95i	2.02c	0.877d	2.897c	20.84f	2.420 cd	1.132e	3.652d
	M	19.17de	2.12c	1.5ab	3.456b	22.22e	2.34d	1.578bc	4.23c
Diseased plants+ Chitosan NPs	NM	18.45ef	2.192abc	1.37ab	3.561b	23.31bc	2.706abc	1.93ab	4.635ab
	M	22.21a	2.483a	1.91a	4.393a	25.003a	3.14ab	2.081ab	5.221a
Diseased plants+ Chitosan	NM	16.91 h	2.177abc	1.3bc	3.473b	22.06e	2.462 cd	1.52 cd	4d
	M	19.87 cd	2.49a	1.571ab	4.058a	23.05 cd	2.861ab	1.907ab	4.77ab
Diseased plant + Fungicide	NM	15.96i	2.177abc	1.3bc	3.456b	22.16e	2.34d	1.52 cd	4d
	M	19.17de	2.483a	1.471ab	4.168a	23.31bc	2.861ab	1.938ab	4.627ab

LSD: At significant level (P *>* 0.05).Sample symbols (a.a) mean non-significant difference (a.b) mean significant difference. NM; Non-mycorrhizal plants and M; Mycorrhizal plants. Chl a: Chlorophyll a ; Chl.b :Chlorophyll b

**Table 5:** The Effect of mycorrhizal infection with different treatments on Phenols content, POX (peroxidase) and PPO. (Polyphenoloxidase) in *Vicia faba* infected with *R. solani*

		After 35 days of sowing			After 70 days of sowing		
		Phenols content mg100 g fw^− 1^	(peroxidase) POX ∆absorbance unit min^− 1^ g fw^− 1^	Polyphenoloxidase ∆absorbance unit min^− 1^ g fw^− 1^	Phenols content mg100 g fw^− 1^	(peroxidase) POX ∆absorbance unit min^− 1^ g fw^− 1^	Polyphenoloxidase ∆absorbance unit min^− 1^ g fw^− 1^
Healthy Plants	N M	20j	6.95e	4.38f	16.7 g	5.74 g	3.53efg
	M	34.9e	9.4d	5.22e	34.32d	7.04ef	4.33bcd
Diseased Plants	N M	31.95f	13.01b	5.85c	23.503e	9.42c	4.95ab
	M	50.7a	14.05a	6.79a	47.82a	11.38a	5.29a
Diseased plants+ Chitosan NPs	NM	28.82 h	9.54d	5.11e	22.13f	7.94de	3.26 fg
	M	47.21b	13.90a	6.56a	43.21b	10.65ab	4.92ab
Diseased plants+ Chitosan	N M	25.15i	8.84d	4.62f	21.77f	6.267 fg	3.13 g
	M	45.34 cd	13.01b	5.6 7 cd	41.24c	10.59ab	4.14cde
Diseased plant + Fungicide	NM	28.82 h	9.54d	4.67f	21.84f	7.91de	3.26 fg
	M	45.34 cd	13.90a	6.56a	41.26c	10.62ab	4.87ab

LSD: At significant level (P *>* 0.05).Sample symbols (a.a) mean non significant difference (a.b) mean significant difference. NM; Non-mycorrhizal plants and M; Mycorrhizal plants. The reading of phenol contents was calculated in mg catechol equivalent of phenol per 100 g fresh weight. Standard curve was prepared using different concentrations of catechol

**Table 6:** Experimental treatments design for growth of *Vicia faba*

	Treatments design	
1	healthy plants	non-inoculated with AMF (NM) Inoculated with AMF (M)
2	Diseased plants with *R.solani*	NM M
3	Diseased plants + Chitosan NPs	NM M
4	Diseased plants + Chitosan	NM M
5	Diseased plants + Fungicide (Rizolex)	NM M

### Estimation of mycorrhizal root colonization and mycorrhizal dependency

Root colonization Frequency; percentage of root segments colonizes (F%) of VF infected with *R. solani* was studied with response to different treatments following the staining of root samples with 0.5% trypan blue in lactophenol method. Randomly stained root segments (1 Cm in length) were examined microscopically with gridline intersect slide method for calculation the percentage of frequency infection (F%) for mycorrhizal roots [62, 63]. Mycorrhizal dependency (MD %); the degree to which a plant is dependent on the mycorrhizal condition in order to produce the maximum growth or yield was determined with the procedure of Menge et al. [64]

### Estimation of growth parameters

Fresh plant samples were taken after 35 & 70 days from sowing of infected and non-infected soil of both non mycorrhizal and mycorrhizal faba bean plants, washed carefully under water to eliminate soil residues and estimated morphological characters as root, shoot length and measuring the dry weight [17].

### Plant macronutrient contents analysis

In each replicate after 35 and 70 days of sowing, three samples of plant shoot were dried at 70^0^ C for 48 h. and finally ground. The digestion of samples was described by Peterburgski, [65]. After digestion, the solution was transferred into 100 mL flask with distilled water then estimate nitrogen, phosphorus and potassium was estimated. Nitrogen was estimated by semi-micro-kjeldahle method [66]. Phosphorus was estimated using a colorimetric technique [67]. Potassium was determined by flame photometry using corning, 400 flame photometer [65]; 1.0 mL from sample + 9.0 mL distilled water and the reading were taken on flame photometer by using red filter.

### Physiological and biochemical parameters

Randomly selected samples of three plants from each pot of each treatment after 35 and 70 days of planting were collected to estimate the nutrients and photosynthetic plant pigments. The pigments were estimated using methanol 90% after adding a traces of sodium carbonate and stored in a dark place then the extract measured with a spectrophotometer at 452.5, 650 and 665 nm with the formula adapted previously [68, 69].Total soluble sugar (T.S. S) content was estimated according to Bing et al. [70].

### Total phenolic compounds

After 35 and 70 days of planting, samples of 0.5 g grounded fresh leaves was putted into centrifuge tube, 5 mL of methanol as solvent (70%) was added, and the sample was mixed for 15 min by shaking, and then centrifuged at 3.000 rpm for 15 min, the pure supernatant was collected. The supernatants were dried. The residue was re-extracted twice dissolved in 5mL of 70% methanol. These supernatants were collected together before removal of methanol and the concentrated extract was dried and stored for further use [71]. The amount of phenolic contents was estimated using Folin-Ciocalteu reagent according to Singleton and Rossi [72].

### Assay of some defense enzymes activities

Fresh leaves samples were collected, weighed and washed after 35 and 70 days from the same pot to measure peroxidase and polyphenol oxidase efficiency; enzymes extract were carried out at 4ºC. Peroxidase enzyme (POX) measurement was prepared using the procedure published with Maxwell and Bateman [73]. Polyphenoloxidase (PPO) enzyme was extracted and assayed with Maria et al. [74].

### Statistical analysis

Information of these studies was analyzed by statistical analysis system [75]. All comparisons were submitting to procedure of variance (ANOVA), with Duncan’s multiple procedures according to. LSD: (P > 0.05).model signs (a.a): non-significant difference (a.b): significant difference [17, 76]. Principal component analysis (PCA) was proceeded on different parameters of VF plants over with *R.solani* in reaction to processing by AMF and Chitosan NPs [77].

## Data Availability

The information introduced in this investigation is available in the article.

## Abbreviations

VF: *Vicia faba*
AMF: Arbuscular mycorrhizae fungi
DI %: Disease Inidence
DS %: Disease Severity
Dwt: Dry weight
MD: Mycorrhizal dependency
F %: mycohrrhizal infection frequency
Chitosan NPs: chitosan nanoparticles
TPP: Tri poly phosphate
DLS: Dynamic light scattering
FTIR: Fourier transform infrared
T.S.S.: Total soluble sugar
POX: Peroxidase
PPO: Polyphenol Oxidase
